# *ATP1A3* Mutations and Genotype-Phenotype Correlation of Alternating Hemiplegia of Childhood in Chinese Patients

**DOI:** 10.1371/journal.pone.0097274

**Published:** 2014-05-19

**Authors:** Xiaoling Yang, Hua Gao, Jie Zhang, Xiaojing Xu, Xiaoyan Liu, Xiru Wu, Liping Wei, Yuehua Zhang

**Affiliations:** 1 Department of Pediatrics, Peking University First Hospital, Beijing, People's Republic of China; 2 Center for Bioinformatics, State Key Laboratory of Protein and Plant Gene Research, School of Life Sciences, Peking University, Beijing, People's Republic of China; 3 National Institute of Biological Sciences, Beijing, People's Republic of China; Shenzhen Institutes of Advanced Technology, China

## Abstract

Alternating hemiplegia of childhood (AHC) is a rare and severe neurological disorder. *ATP1A3* was recently identified as the causative gene. Here we report the first genetic study in Chinese AHC cohort. We performed whole-exome sequencing on three trios and three unrelated patients, and screened additional 41 typical cases and 100 controls by PCR-Sanger sequencing. *ATP1A3* mutations were detected in 95.7% of typical AHC patients. At least 93.3% were *de novo*. Four late onset, atypical AHC patients were also mutation positive, suggesting the need for testing *ATP1A3* mutations in atypical cases. Totally, 13 novel missense mutations (T370N, G706R, L770R, T771N, T771I, S772R, L802P, D805H, M806K, P808L, I810N, L839P and G893R) were identified in our study. By homology modeling of the mutant protein structures and calculation of an extensive list of molecular features, we identified two statistically significant molecular features, solvent accessibility and distance to metal ion, that distinguished disease-associated mutations from neutral variants. A logistic regression classifier achieved 92.9% accuracy by the average of 100 times of five-fold cross validations. Genotype-phenotype correlation analysis showed that patients with epilepsy were more likely to carry E815K mutation. In summary, *ATP1A3* is the major pathogenic gene of AHC in Chinese patients; mutations have distinctive molecular features that discriminate them from neutral variants and are correlated with phenotypes.

## Introduction

Alternating hemiplegia of childhood (AHC, OMIM 614820) is a rare and severe neurological disorder [1]. AHC occurs mostly in sporadic cases, though familial cases have been reported [2], [3]. It is characterized by episodic hemiplegia or quadriplegia attacks, accompanied by other paroxysmal symptoms, including oculomotor abnormalities, dystonia, seizures and autonomic disturbances. The age of onset is usually before 18 months [4], [5]. Most patients also display developmental delay and progressive cognitive impairment. AHC was initially regarded as a hemiplegic migraine variant, but genes responsible for familial hemiplegic migraine such as *CACNA1A*, *ATP1A2* and *SLC1A3* have failed to be confirmed as causative for AHC [6]–[8]. Sodium-potassium (Na^+^/K^+^) ATPase α3 subunit (*ATP1A3*) has recently been identified as a causal gene for sporadic AHC by three groups [2], [9], [10], resulting in 82.2% positive rate and at least 78.9% as *de novo* for European/American samples. However there had been no large genetic study of Chinese cohort. Such a study may not only confirm the main causal gene in Chinese patients, but also result in the discovery of novel mutations.

The protein encoded by *ATP1A3* is a subunit of an integral membrane protein responsible for maintaining the sodium and potassium concentration gradients. It has two structural conformations, E1 and E2 that selectively bind three Na^+^ and two K^+^ respectively [11]. The E2 structure of its homologous protein has been resolved in 2007 [11], and recently E1 structure has also been resolved [12]. The protein crystal structures show that ion binding sites locate inside transmembrane helixes M4∼M8. The mutation D801N, demonstrated by Heinzen *et al*. [2], apparently prevents the binding of K^+^ at E2 conformation. But other variants are not so close to the ion binding site and their influences on protein functions remain unclear. An extensive survey of the molecular features of the mutations may shed light on the etiology of diseases and be useful for predicting the pathogenicity of novel *ATP1A3* variants.

Besides association with AHC, *ATP1A3* was first reported to be associated with rapid-onset dystonia-parkinsonism (RDP, OMIM 128235), a distinctive autosomal-dominant movement disorder [13]. Rosewich *et al* noted that AHC and RDP may make up a continuum of a dystonic movement disorder, but they also have a considerable list of different characteristics [9]. Furthermore, *ATP1A3* mutations identified in AHC and RDP patients so far have no overlap except for one mutation D923N [3], [14], [15]. This implied that different mutation in *ATP1A3* may give rise to different phenotype. Finally, there is a need to investigate the genotype-phenotype correlations of AHC, which would be valuable in clinical diagnosis.

## Materials and Methods

### Standard protocol approvals, registrations, and patient consents

This study was approved by Institutional Review Boards at Peking University First Hospital. Written informed consent was obtained from all participants or their parents in case of minors. The diagnosis of all the patients was made according to clinical diagnostic criteria for typical AHC as follows [4], [16]: (1) onset of paroxysmal events before 18 months of age, (2) repeated bouts of hemiplegia involving right and left side of the body in some attacks, (3) episodes of bilateral hemiplegia or quadriplegia starting either as generalization of a hemiplegic episode or bilateral from the start, (4) other paroxysmal disturbances including tonic/dystonic attacks, nystagmus, strabismus, dyspnoea and other autonomic phenomena occurring during hemiplegic bouts or in isolation, (5) immediate disappearance of all symptoms upon sleep, with probable recurrence of long-lasting bouts 10–20 min after awakening, (6) evidence of developmental delay, mental retardation, neurologic abnormalities, choreoathetosis and dystonia or ataxia, (7) not attributable to other disorders. When age of onset was later than 18 months but patients fulfilled the other AHC diagnostic criteria, these patients were considered as atypical cases [17]. A total of 51 AHC patients were enrolled including a monozygotic twin which were counted as one patient. 47 of them fulfilled the diagnostic criteria for typical AHC. Four patients were considered as atypical cases, since their age of onset was later than 18 months. Venous blood samples were obtained from the participants and their parents. Detailed clinical phenotypes including the disease onset, initial symptoms, paroxysmal and non-paroxysmal manifestations, EEG and magnetic resonance imaging (MRI) results were acquired by face-to-face interviews, questionnaires, and telephone follow-up.

### Whole-exome sequencing

Genomic DNA was extracted from venous blood according to standard protocol from three trios and three sporadic cases. Exomes were captured by Agilent SureSelect Human All Exon 50 Mb Kit and sequenced on Illumina HiSeq 2000. Sequence analysis was performed following the best practice of GATK v1.6 [18]. Variants that fit the *de novo* dominant model were selected for further functional annotations and filtered following the ANNOVAR protocol [19]. Local assembly was performed to handle structural variations and insertions/deletions [20]. A gene was reported as a candidate gene if it had a *de novo* functional mutation (including insertion/deletion, splicing, nonsense and missense mutation) in at least one of the trios and has a functional or missense mutation in at least one other unrelated case. *ATP1A3* was confirmed as the only candidate gene.

### Sanger sequencing

Mutations identified by next-generation sequencing were validated by PCR-Sanger sequencing. Primer sequences were the same as that published in previous work [9]. The remaining unrelated patients were sequenced on all 23 exons of *ATP1A3*. Mutation sites were sequenced in parents to determine whether the mutations were transmitted or *de novo*. 100 unrelated healthy individuals were sequenced as normal controls.

### Statistical analysis of molecular features of the variants

Variants in *ATP1A3* were collected from our data, published genetic studies of AHC and RDP, HGMD database [21], 1000 Genomes Project [22] and NHLBI Exome Sequencing Project [23]. For each missense variant, we took the crystal structure of pig *ATP1A1* as reference (identity 86.2%, similarity 93.2%). We modeled its mutant protein 3D structure at both E1 [12] and E2 [11] conformations by homology modeling with SWISS-MODEL [24] followed by energy minimization [25]. An extensive list of molecular features was calculated by SAPred [26], PyMOL and other tools (see “Web resources” section). For each molecular feature, its correlation with neutral *versus* disease-associated status, and AHC-associated *versus* RDP-associated, were calculated using Spearman's rho and term's *P*-value in logistic regression in the R software. Lasso [27] was employed for robust feature selection: 10000 times of bootstrap produced 10000 simulation datasets; in each dataset Lasso selected discriminative features as few as possible; the number of times each feature was chosen was recorded and ranked. Two of the most significantly correlated features were selected to build a logistic regression classifier. The decision boundary of classifier was further verified in consideration of weight. Frequency weight was set according to how many individuals carried the variant. Precision weight for mutation hotspot, recurrent mutation and singleton mutation were set as 2, 1 and 0.75 respectively. The third strategy used all mutations for training without weighting. The accuracy of the classifier was assessed by 100 times of five-fold cross validations.

### Genotype-phenotype correlation analysis

Clinical data of the AHC patients were collected from our own Chinese cohort as well as the reports by Heinzen *et al*. [2] and Rosewich *et al*. [9]. Potential population differences in *ATP1A3* mutations between Chinese and European/American were assessed. The mutation frequencies of two populations were compared using Fisher's exact test and the population differentiation was measured by F_st_ based on data from HapMap Project [28]. The incident rate of each symptom was also compared. Symptoms that every patient would have were excluded from our analysis, such as hemiplegia and abnormal eye movement. The three mutation hotspots were analyzed to find correlations with each symptom and with the Flunarizine treatment effect by Fisher's exact test. Multiple hypothesis testing correction was done by FDR correction [29].

### Web resources

The SAP disease-association predictor [26] (SAPred; http://sapred.cbi.pku.edu.cn/) is an automatic pipeline to predict the disease-associated single amino acid polymorphism and takes an extensive list of molecular features into account. In this study we used its intermediate feature matrix.

FoldX [30] (http://foldx.crg.es/) was used to estimate protein stability based on energy change.

PoPMuSic [31] (http://babylone.ulb.ac.be/popmusic) was used to estimate protein stability and solvent accessibility.

SCPred [32] (http://www.enzim.hu/scpred) was used to estimate protein stability based on prediction of residues in stabilization centers.

DisEMBL [33] (http://dis.embl.de) was used to predict whether a mutation is located in disordered regions.

ProtScale [34] (http://web.expasy.org/protscale) was used to view the crystal structure and calculate distances between atoms.

PyMOL (http://www.pymol.org/) was used to view the crystal structure and calculate distances between atoms.

## Results

### Genetic findings

We recruited 47 patients fulfilled the diagnostic criteria for typical AHC. The male to female ratio was 1∶0.62 (Table 1). Whole-exome sequencing of three trios and three sporadic cases produced about 18.8 Giga bases of raw sequence data per individual. 98.1% of raw reads could be properly mapped to human reference genome. On average, each base in the capture region has been sequenced about 200X. Following our analysis pipeline, rare mutations in *ATP1A3* were identified in all six patients. All mutations were missense and validated by further Sanger sequencing. Together with Sanger sequencing of all *ATP1A3* exons in other 41 typical AHC patients, 95.7% (45/47) patients were found to carry *ATP1A3* mutations. In total, nineteen missense mutations (at nucleotide level, Table 2) were identified. Eight of them had been reported in AHC cases before, and eleven were novel mutations reported for the first time. These novel mutations were located at highly conserved sites (Figure S1) and were absent in 100 normal controls, the public 1000 Genomes databases and NHLBI Exome Sequencing Project. These data confirmed *ATP1A3* as the main causal gene in the Chinese cohort.

**Table 1:** *ATP1A3* mutations and clinical features of patients with alternating hemiplegic of childhood.

ID	Sex	Nucleotide change	Amino acid change	Father	Mother	Age at lastassessment	Age of onset	First symptom	Hemiplegic (onset age, duration, frequency)	Quadriplegia	Abnormal eye movement	Dystonia	Epilepsy	Developmental delay	Treatment effect of flunarizine
A02103	F	c.410C>A	S137Y	−	−	3 y9 m	2 m	abnormal eye movement	2 m, 1∼3 d, 1∼3 per m	+	+	+	−	+	−
A01903	F	c.1109C>A	T370N	−	−	11 y11 m	12 m	hemiplegia	12 m, 3∼4 d, NA	+	+	+	−	+	NA
A05003	M	c.2116G>A	G706R	−	−	8 y	2 m	abnormal eye movement	38 m, 1 h∼7 d, 4∼5 per m	+	+	+	−	+	+
A03203	M	c.2263G>A	G755S	−	−	1 y4 m	3 m	abnormal eye movement	7 m, 2 d, 4∼7 per m	−	+	+	−	+	+
A06403	M	c.2263G>T	G755C	−	−	4 y1 m	1.5 m	abnormal eye movement	10 m, 1∼5 d, 2∼3 per m	+	+	+	−	+	Untreated
A02603	M	c.2312C>A	T771N	−	−	6 y2 m	1.5 m	abnormal eye movement	7 m, 0.5 h∼2 d, 2∼3 per m	−	+	+	−	+	+
A00603	M	c.2312C>T	T771I	−	−	14 y3 m	10 m	hemiplegia	10 m, 0.1 h∼5 d, 2 per m	+	+	+	−	+	NA
A03603	M	c.2316C>G	S772R	−	−	6 y1 m	4 m	dystonia	10 m, 3∼4 d, 2∼10 per m	+	+	+	−	+	−
A01103	F	c.2401G>A	D801N	−	−	5 y6 m	5 m	abnormal eye movement	5 m, 4∼5 d, 2∼4 per m	+	+	+	−	+	−
A02003	F	c.2401G>A	D801N	−	−	3 y6 m	1 m	abnormal eye movement	4 m, 2∼3 d, 2 per m	+	+	+	−	+	+
A02403	M	c.2401G>A	D801N	−	−	2 y9 m	2 m	hemiplegia	2 m, 2∼3 d, 2 per m	+	+	+	−	+	−
A02203	M	c.2401G>A	D801N	−	−	8 y	4 m	hemiplegia	4 m, 2∼4 d, 1∼4 per m	+	+	−	−	+	−
A02503	F	c.2401G>A	D801N	−	−	6 y	7 m	hemiplegia	7 m, 3∼5 d, 2 per m	−	+	−	−	+	−
A03103	M	c.2401G>A	D801N	−	−	1 y9 m	4 m	quadriplegia	4 m, 3 d, 1∼3 per m	+	+	+	−	+	+
A04203	F	c.2401G>A	D801N	−	−	3 y7 m	10 m	hemiplegia	10 m, 3∼5 d, 3∼10 per m	+	+	+	−	+	−
A03503	M	c.2401G>A	D801N	−	−	1 y6 m	2.5 m	dystonia	6 m, 1∼2 h, 1∼3 per m	+	+	+	−	+	+
A02803	M	c.2401G>A	D801N	−	−	2 y5 m	2 m	dystonia	2.3 m, 3∼5 d, 1∼2 per m	+	+	+	−	+	+
A03703	M	c.2401G>A	D801N	−	−	9 m	2.5 m	abnormal eye movement	5.3 m, 6 h∼2 d, 1∼2 per m	+	+	+	−	+	+
A03903	M	c.2401G>A	D801N	−	−	11 y11 m	3 m	hemiplegia	3 m, 3∼4 d, 8∼10 per m	+	+	+	−	+	+
A04603	F	c.2401G>A	D801N	−	−	1 y3 m	3.5 m	abnormal eye movement	6 m, 2∼3 d, 3∼4 per m	+	+	+	−	+	+
A04803	M	c.2401G>A	D801N	−	−	1 y5 m	3 m	abnormal eye movement	6 m, 2∼3 d, 2∼3 per m	+	+	+	−	+	−
A06103	M	c.2401G>A	D801N	−	−	4 y9 m	1 m	abnormal eye movement	1 m, 4∼7 d, 3∼4 per m	+	+	+	−	+	−
A05603	M	c.2405T>C	L802P	−	−	7 y	14 m	abnormal eye movement	14 m, 1 h∼7 d, 3∼4 per m	+	+	−	−	+	+
A05604	M	c.2405T>C	L802P	−	−	7 y	8 m	abnormal eye movement	14 m, 3∼4 d, 3∼4 per m	+	+	−	−	+	+
A06203	F	c.2413G>C	D805H	−	−	4 y3 m	2 m	abnormal eye movement	8 m, 2∼3 d, 3∼10 per m	−	+	+	−	+	+
A00303	M	c.2417T>A	M806K	−	NA	7 y6 m	3 m	abnormal eye movement	10 m, 1∼7 d, 1 per m	−	+	+	−	+	+
A05403	M	c.2423C>T	P808L	−	−	1 y9 m	4 m	abnormal eye movement	7 m, 2 h∼7 d, 3∼4 per m	+	+	+	−	+	−
A00403	M	c.2429T>A	I810N	−	−	12 y1 m	1.5 m	abnormal eye movement	6 m, 1∼3 d, 1∼10 per m	+	+	+	+	+	+
A00703	M	c.2443G>A	E815K	−	−	6 y6 m	6 m	hemiplegia	6 m, 1∼4 d, 1∼4 per m	−	+	+	+	+	+
A00503	F	c.2443G>A	E815K	−	−	10 y9 m	6 h	abnormal eye movement	7 m, 3∼5 d, 1∼4 per m	−	+	+	+	+	NA
A01303	M	c.2443G>A	E815K	NA	−	4 y7 m	1 m	dystonia	4 m,2 d, 3∼4 per m	−	+	+	−	+	−
A01503	F	c.2443G>A	E815K	−	−	3 y10 m	4 m	seizure	4 m, 0.5 h∼1 d, 3∼4 per m	+	+	+	+	+	+
A03303	M	c.2443G>A	E815K	−	−	1 y10 m	1 m	dystonia	17 m, 2∼3 d, 1 per m	−	+	+	+	+	+
A04003	M	c.2443G>A	E815K	−	−	2 y	1 m	abnormal eye movement	4.5 m, 2∼5 d, 1∼2 per m	−	+	+	−	+	+
A02303	M	c.2443G>A	E815K	−	−	4 y3 m	1 m	abnormal eye movement	4 m, 2∼3 d, 4 per m	−	+	+	+	+	+
A06003	F	c.2443G>A	E815K	−	−	4 y3 m	9 m	seizure	9.5 m, 1∼3 d, 3∼10 per m	+	+	+	+	+	+
A06303	M	c.2443G>A	E815K	−	−	4 y11 m	2 m	dystonia	7 m, 3∼7 d, 2∼6 per m	+	+	+	+	+	+
A05903	F	c.2516T>C	L839P	−	−	1 y5 m	8 m	abnormal eye movement	8 m, 0.5 h∼7 d, 1∼2 per m	+	+	+	−	+	+
A02703	F	c.2767G>T	D923Y	−	−	4 y11 m	2 m	abnormal eye movement	18 m, 1∼4 d, 1∼2 per m	−	+	+	−	+	NA
A00103	M	c.2839G>A	G947R	−	−	20 y10 m	2 d	abnormal eye movement	36 m, 8∼9 d, 3∼4 per m	+	+	+	−	+	+
A00203	F	c.2839G>A	G947R	−	−	10 y6 m	4 m	abnormal eye movement	7 m, 0.5 h∼5 d, 2∼3 per m	−	+	+	−	+	−
A01603	M	c.2839G>A	G947R	−	−	6 y	12 m	hemiplegia	12 m,7 d, 4 per m	−	+	+	−	+	+
A03003	F	c.2839G>A	G947R	−	−	2 y5 m	1.5 m	abnormal eye movement	4.5 m, 1∼5 d, 4 per m	+	+	+	−	+	+
A05303	F	c.2839G>A	G947R	−	−	8 y8 m	4.5 m	abnormal eye movement	5 m, 0.5 h∼3 d, 3∼4 per m	+	+	+	−	+	+
A02903	F	c.2839G>C	G947R	−	+	1 y6 m	5 m	dystonia	12 m, 1∼2 d, 2∼3 per m	+	+	+	−	+	+
A05503	M	c.2839G>C	G947R	−	−	4 y11 m	7 m	abnormal eye movement	7 m, 0.5 h∼3 d, 2∼3 per m	+	+	+	−	+	−
A05803	M	−	−	−	−	3 y6 m	7 m	dystonia	7 m, 1∼5 h, 0∼1 per m	−	+	+	−	−	Untreated
A04503	F	−	−	−	−	4 y6 m	3.5 m	abnormal eye movement	23 m, 0.5∼1 h, 2∼30 per m	+	+	−	−	−	+
A01203	M	c.2839G>C	G947R	−	−	17 y6 m	30 m	abnormal eye movement	30 m, 0.5 h∼4 d, 4∼7 per m	+	+	+	−	+	−
A01403	M	c.2309T>G	L770R	−	+	9 y5 m	42 m	hemiplegia	43 m, 0.1∼0.5 h, 0∼7 per d	+	−	−	−	+	Untreated
A04103	M	c.2767G>A	D923N	−	−	3 y6 m	30 m	quadriplegia	30 m, 3 h∼7 d, 4∼7 per m	+	+	+	−	+	+
A05203	F	c.2677G>A	G893R	−	−	7 y3 m	24 m	hemiplegia	24 m, 0.2 h∼5 d, 2∼3 per m	−	+	−	−	+	−

F: female, M: male, y: year, h: hour, d: day, m: month, +: positive, −: negative, NA: not available, Patients A05603 and A05604 were two monozygous twins.

**Table 2:** *ATP1A3* mutations identified in 45 Chinese typical AHC patients.

Nucleotide change	Exon	Amino acid change	Number of cases
c.410C>A	5	S137Y	1
c.1109C>A^a^	9	T370N	1
c.2116G>A^a^	16	G706R	1
c.2263G>A	16	G755S	1
c.2263G>T	16	G755C	1
c.2312C>A^a^	17	T771N	1
c.2312C>T^a^	17	T771I	1
c.2316C>G^b^	17	S772R	1
c.2401G>A	17	D801N	14
c.2405T>C^a^	17	L802P	1
c.2413G>C^a^	17	D805H	1
c.2417T>A^c^	17	M806K	1
c.2423C>T^a^	18	P808L	1
c.2429T>A^c^	18	I810N	1
c.2443G>A	18	E815K	9
c.2516T>C^a^	18	L839P	1
c.2767G>T	20	D923Y	1
c.2839G>A	21	G947R	5
c.2839G>C	21	G947R	2

*ATP1A3* mutation coordinates were defined on the basis of NM_152296.4 and NP_689509.1.

a The locus of mutation was newly identified.

b The locus of mutation and amino acid change had been reported before, but nucleotide change was newly identified.

c The locus of mutation had been reported before, but nucleotide change and amino acid change were newly identified.

Three mutation hotspots, D801N, E815K and G947R (which contained two mutations at nucleotide level: c.2839G>A and c.2839G>C), were detected in 31.1% (14/45), 20.0% (9/45) and 15.6% (7/45) *ATP1A3* positive patients, respectively (Table 2). *De novo* rate was at least 93.3% (42/45), and could be as high as 97.7% (42/43) because the blood of two probands' parents was not available (Table 1). Only one patient (A02903) was found to inherit G947R mutation from her mother who was also affected with AHC (Figure S2 and Discussion).

Besides, there were four atypical AHC cases in our cohort whose age at onset was later than 18 months. They all carried missense mutations in *ATP1A3* (Table 1) too. Patient A01203 carried a *de novo* hotspot mutation G947R, while patient A05203 carried a novel and *de novo* mutation G893R. Unexpectedly, patient A04103 carried a *de novo* mutation D923N which was previously reported to be associated with RDP and in an AHC family [3], [14], [15], and patient A01403 inherited a novel mutation L770R from his unaffected mother. All these mutations were in conserved loci and absent in 100 normal controls and public 1000 Genomes data and NHLBI Exome Sequencing Project data.

The sequencing data had been submitted to dbGaP with accession number phs000660.v1.p1. All the mutations had been deposited in the LOVD database (www.lovd.nl/ATP1A3) and dbSNP.

### Discriminative molecular features

**Figure 1:**
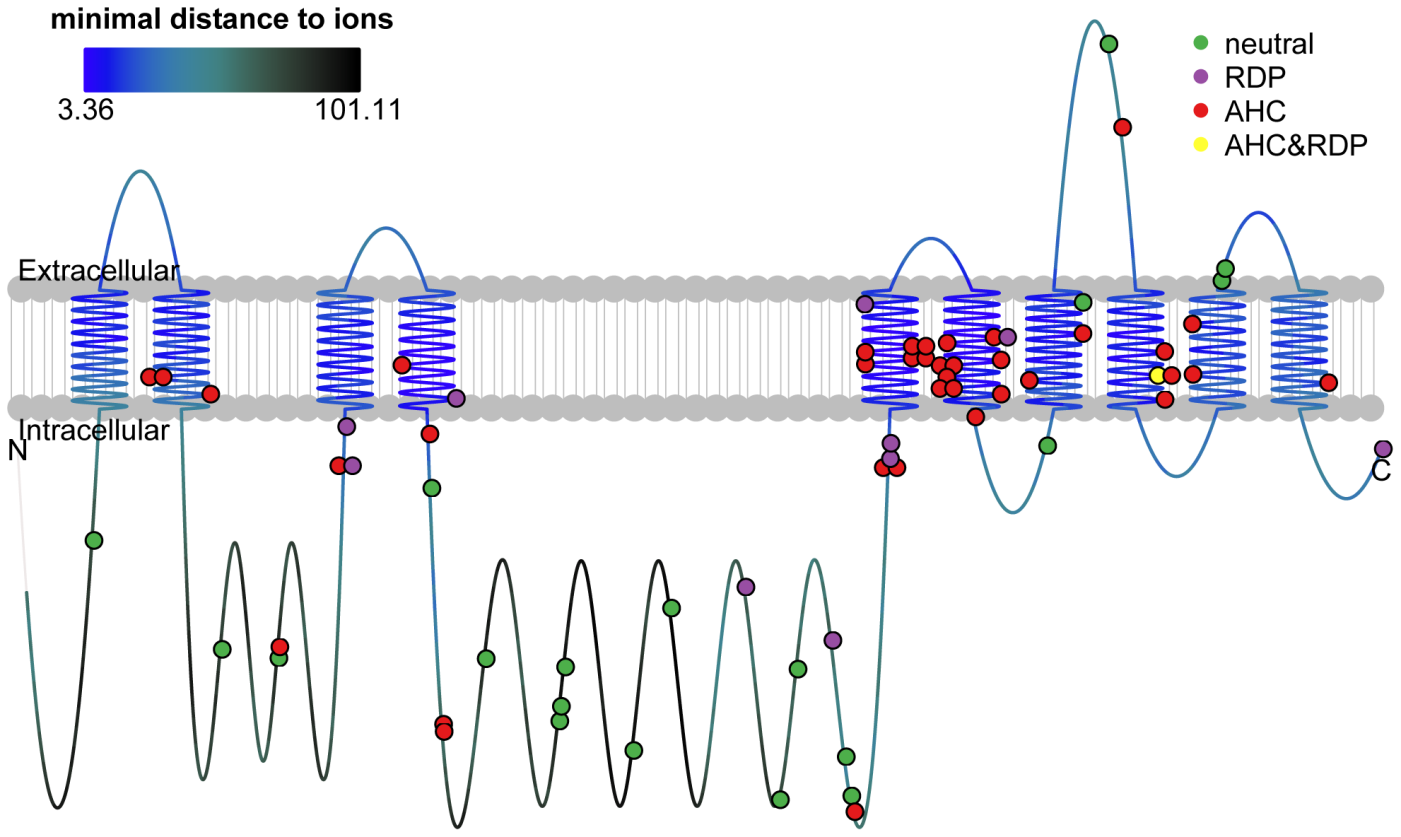
Locations of *ATP1A3* variants and mutations shown on protein domains. The line represented the protein and the colors of the line represented distance to metal ion binding sites, as the upper left panel showed. The dots represented variants and mutations (for insertion/deletion, the dot marked the beginning position) with the colors representing phenotype categories, as the upper right legend showed. The raw data for this figure was in Table S1.

In addition to mutations identified in our own cohort, we collected all previously reported mutations in *ATP1A3* in AHC patients and RDP patients, as well as variants in normal individuals (neutral variants) (Table S1). We found no overlap among the three groups of variants except for D923N which was first reported in RDP patients [14], [15] and recently in familial AHC patients [3] as well as in one of our atypical patients (Patient A04103). When viewed on top of the protein domains (Figure 1 and Table S1), there was clear difference (Fisher's exact test *P*-value <0.001). Mutations associated with AHC were predominantly located in transmembrane domains (73.7% of AHC-associated mutations were located in transmembrane domains, compared to 13.8% of those not associated, *P*-value <0.001), especially αM5 and αM6 which were nearest to metal ion binding sites [11]. Most neutral variants were away from transmembrane domains (5.3% compared to 64.6%, *P*-value <0.001). In contrast, mutations associated with RDP showed no location bias (36.4% compared to 50.0%, *P*-value 0.517).

Further, we built 3D structure models of mutant proteins in both E1 and E2 conformations for each missense mutation/variant (18 of them classified as neutral variants, 35 as AHC-associated, 8 as RDP-associated and 1 as both AHC and RDP associated; Table S2) and calculated 71 molecular features including protein aggregation property, amino acid composition, conservation, distance to metal ion site, secondary structure, solvent accessibility, protein stability and stereo-chemistry property (Table S2).

**Figure 2:**
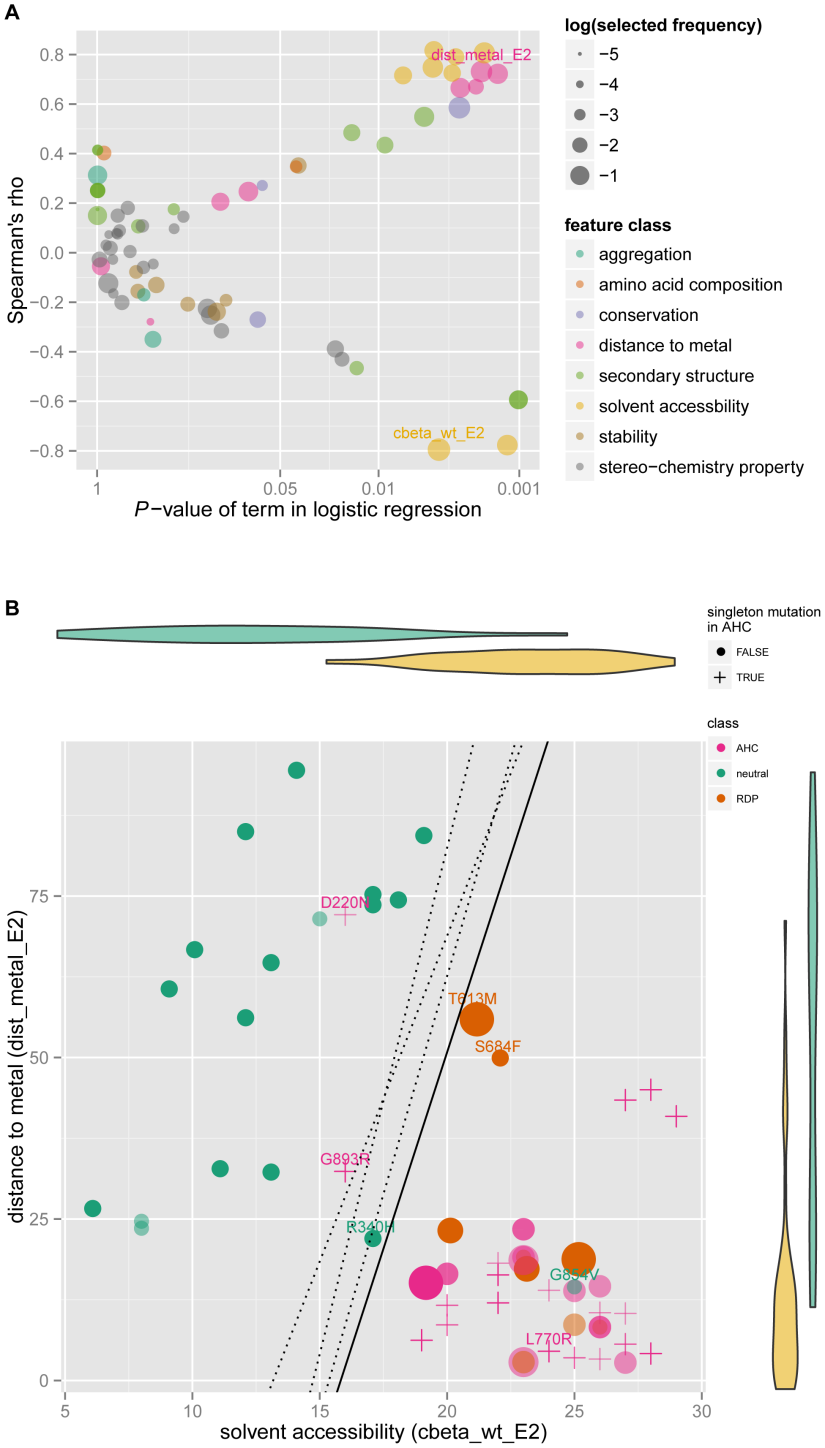
Discriminative features and classifier for disease-associated mutations *versus* neutral variants. (A) Discriminative effect and correlation of each molecular feature based on training dataset. X-Y axes demonstrated correlation between each feature with variant category through logistic regression analysis and Spearman's rho calculation. The dot color represented the class the molecular feature belonged to, and size meant selected frequency in robust feature selection procedure. The selected features were labeled. (B) Classifiers and its prediction result. X-Y axes represented the selected features ‘cbeta_wt_E2’ (the number of β carbon atoms around the mutated site within 10 Å in E2 wildtype protein structure) and ‘dist_metal_E2’ (the minimum distance from mutated site to metal ion binding pocket in E2 wildtype protein structure; unit Å), belonging to ‘solvent accessibility’ and ‘distance to metal site’ class respectively. The violin diagrams demonstrated the distribution of each feature in each variant category. The crosses indicate singleton mutations in AHC, while dots were mutations used in the train dataset. The size of the dots represented precision weights. The solid line was corresponded to the simplest model without any weight, while the three dotted lines from left to right according to the intercept on X axis were the decision boundaries of three different models (see Methods section): using all mutations for training; weighting train dataset with frequency weight; weighting train dataset with precision weight.

Among AHC-associated missense mutations, there were twenty-three singleton mutations, seven of which were reported by Heinzen *et al*. [2], three by Rosewich *et al*. [9], one by Ishii *et al*. [10] and twelve in our study. Given the lack of recurrence, to ensure quality we excluded them from the classifier building procedure. Compared to neutral variants, in addition to the tendency to be located in transmembrane regions, the sites of the disease-associated mutations also showed lower solvent accessibility, closer distance to metal ion binding sites and higher conservation. These four classes of features were also frequently selected in robust feature selection procedure by Lasso (Figure 2A, and Table S3A). To minimize the possibility of overfitting, we chose two features which were top of the list of both correlation and selected frequency. They were ‘cbeta_wt_E2’ (the β carbon atoms density around the mutated site within 10 Å in wildtype protein E2 conformation; calculated by SAPred), and ‘dist_metal_E2’ (the minimum distance from mutated site to metal ion binding pocket in wildtype protein E2 conformation; calculated by PyMOL). The higher the value of ‘cbeta_wt_E2’ was and the lower the value of ‘dist_metal_E2’ was, which meant less accessible and closer to metal ion, the more likely the mutation was disease-associated. From the scatterplot (Figure 2B), it was apparent that most disease-associated mutations clustered in the lower right corner. Based on these two features, we built a logistic regression model. The average of 100 times of five-fold cross validation accuracy for this procedure was as high as 92.9% (95% confidence interval on empirical distribution was 71.4%∼100%). Importantly, this classifier predicted almost all singleton missense mutations as disease associated except for D220N and G893R. D220N was reported as *de novo* mutation in one trio by Heinzen *et al*., and G893R was *de novo* in one atypical case in our sample. When we trained other classifiers taking into consideration mutation frequency weights, the decision boundaries shifted around a bit, but the prediction result still held (Figure 2B).

Using the same methods, comparison of AHC-associated mutations *versus* RDP-associated mutations revealed differences between them in terms of transmembrane region and alteration of stereo-chemistry properties (Table S3B), but none of the features reached statistical significance, which could be at least partly due to the small sample sizes.

### Genotype-phenotype correlation

We collected detailed phenotype data from our cohort, including both typical and atypical AHC patients, but the following statistical analyses were only performed on the 47 typical cases. All patients had abnormal eye movement and hemiplegia. Up to 70.2% of the patients developed their first symptom before four months and 40.4% before two months. Abnormal eye movement was the initial symptom in 57.4% of the patients at a median age as early as 2 months, making it the most common and earliest onset.

**Figure 3:**
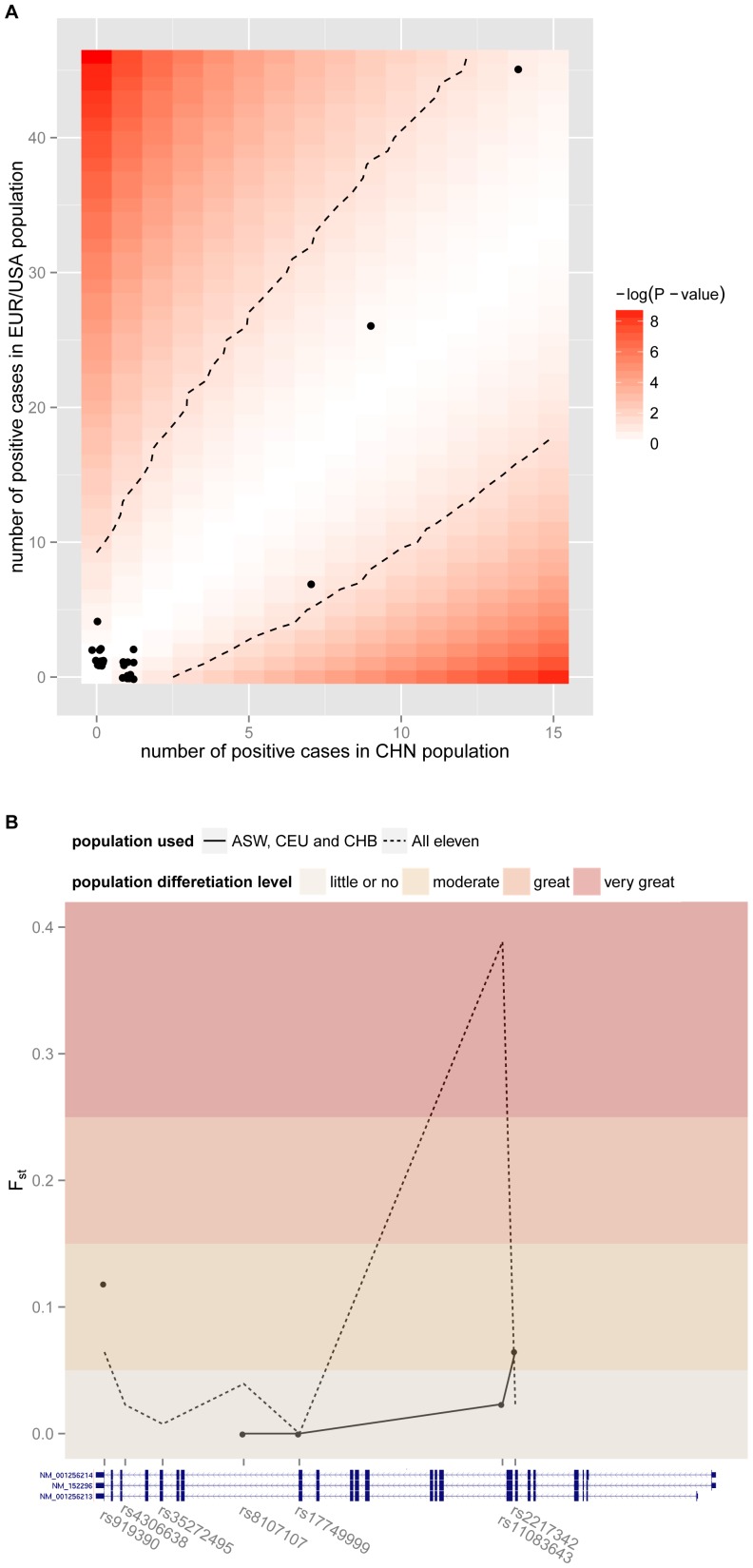
Mutation frequency comparison of *ATP1A3* between Chinese and European/American populations. (A) Mutation frequency comparison of AHC. Each dot represented one mutation, whose value of X axis was the number of cases carrying this mutation out of 47 Chinese AHC cases, and value of Y axis was the number of carriers out of 106 European/American AHC cases. The background showed the *P*-value of Fisher's exact test and dashed line represented 0.05 significant level. (B) Overall population difference of *ATP1A3* according to F_st_ values. The dots and solid line represented F_st_ values of informative SNPs in the populations of Chinese, European and American, and the dotted line represented all eleven populations in HapMap Project. The threshold of F_st_ were set according to S. Wright [40].

Previous genetic studies on AHC were mainly conducted on European/American samples. The genetic architecture of Chinese AHC cohort was first revealed in this study. We assessed the population differences between Chinese and European/American patients. We found that no mutation showed significant difference in allele frequency (Figure 3A), and overall *ATP1A3* gene exhibited no marked population differences (mean F_st_ was 0.04. Figure 3B). However incident rates of two symptoms were different. 71.7% European/American AHC patients exhibited dystonia, while the rate was as high as 91.5% in Chinese (Fisher's exact test *P*-value 0.007); 54.5% European/American AHC patients exhibited epilepsy, while the rate was only 17.0% in Chinese (Fisher's exact test *P*-value <0.001).

A mouse mutant strain *Myshkin* that carries heterozygous I810N mutation in *ATP1A3* has been reported to show generalized seizures [35]. This mutation corresponds to I810N in human *ATP1A3*, and one patient in our cohort (A00403) who carried this mutation did exhibit epilepsy. Unfortunately because of limited sample size positive for I810N, we could not verify the statistical association between epilepsy with I810N. However, investigating the genotype-phenotype correlation for the three mutation hotspots, we found that patients exhibiting epilepsy were more likely to carry E815K mutation (Fisher's exact test *P*-value <0.001, FDR corrected *P*-value 0.010).

Forty-one patients were treated with Flunarizine, 28 of whom showed reduced severity, duration, or frequency of hemiplegic attacks (Table 1) although none had been completely cured. We found no correlation between treatment effects and the three mutation hotpots, which was not surprising because the direct target of Flunarizine was not *ATP1A3*.

### A web site of *ATP1A3* variants and predictions

We set up a freely available website at http://ahc.cbi.pku.edu.cn for continued update of genetic variations and other related information of AHC and *ATP1A3*, and prediction of the functional effects of *ATP1A3* variants.

## Discussion

### *ATP1A3* causes AHC mainly through *de novo* mutations

Our study was the first reported genetic study of AHC in Chinese Han patients. All *ATP1A3* mutations were identified in typical cases as *de novo* except for one familial case. This case came from a three-generation family with two affected individuals (Figure S2). The proband (Patient A02903) inherited G947R mutation from her affected mother. The mother had onset of eye deviation and dystonia at six months. She developed alternating episodes of hemiplegia at one year of age with or without eye deviation and dystonia, at the frequency of once per month, lasting about one hour per episode. She had no seizures. Flunarizine reduced her frequency of hemiplegia to once every half a year and also alleviated symptoms. She had mild developmental delay. She was 26 years old at last follow up, and could take care of herself and do house-work when not having an episode. The grandmother had no neurological symptoms and was confirmed with no mutation at the position. The grandfather had passed away and thus was not available for sequencing, but he had no neurological symptoms. Therefore, the mother's mutation was likely *de novo*.

### Application of the mutation classifier on *ATP1A3*

Given the high cross validation accuracy and stable decision boundary, our classifier would be useful for prediction of whether novel missense variants are neutral or predisposing for AHC or RDP. This method would be applicable to other genetic diseases, especially those without hotspot mutations. From Figure 2B, we noticed that two missense mutations (T613M and S684F) associated with RDP were a little far away from the cluster of disease-associated mutations and interspersed like neutral variants. This agreed with the fact that RDP was less severe than AHC in some aspects such as cognitive development [9]. What's more, G854V which was collected from NHLBI Exome Sequencing Project, was clearly inside the cluster of disease-associated mutations. We suspected that the carrier was susceptible to RDP, but had been free from triggers.

### Genetic testing would facilitate differential diagnosis

AHC often manifested with ocular deviation and dystonia. These early symptoms were often misdiagnosed as epileptic seizures and treated with antiepileptic drugs. Epilepsy may coexist in some AHC patients [16], [36], [37]. Recognizing early clinical features of AHC and video EEG monitoring of episodes are important for differential diagnosis. *ATP1A3* mutation screening can be highly effective for differential diagnosis, especially in the early stages of AHC.

Besides our samples in which *ATP1A3* accounted for 95.7% in the typical AHC cases and 100% in four atypical cases, other studies have reported several atypical patients who also carried *ATP1A3* mutations. Heinzen *et al* reported one AHC family in which an affected individual had onset of episodes of whole body tonic stiffening at three years old and was confirmed to have *ATP1A3* mutation [2]. Roubergue *et al* reported an AHC family with four affected subjects over three generations, including three atypical cases and one typical case. Heterozygous mutation D923N in *ATP1A3* were detected in all four cases [3]. Taken together, these evidences suggested that mutation analysis of *ATP1A3* gene is helpful to confirm the atypical AHC cases.

Given the high positive rate of *ATP1A3* mutations in atypical cases and that one of them (Patient A01203) shared the hotspot mutation G947R, the typical and atypical cases may share the same pathogenesis. It suggested that the diagnostic criteria of onset of paroxysmal events before 18 months of age may need to be relaxed.

### Potential genetic complexity exists

The aforementioned D923N mutation was also discovered in one of the atypical AHC patients in our cohort (Patient A04103). This mutation had previously been reported in two unrelated RDP patients [14], [15], one of whom was atypical for RDP because his age of onset was earlier and he had paroxysmal episodes. Although the vast majority of AHC and RDP mutations had no overlap, this one mutation seemed to cause distinct phenotypes, indicating that other genes or epigenetic and environmental factors may modify the clinical features.

Mutation L770R was discovered in an atypical AHC patient (Patient A01403) and inherited from his unaffected mother. This mutation was located at a highly conserved site and absent from normal controls, and predicted to be highly associated with disease by our classifier with 98.6% probability, implying that it was likely a functional mutation. The mother carried the L770R mutation but had no clinical symptoms of AHC or RDP at last follow up when she was 36 years old. It may be due to incomplete penetrance.

### Development of targeted and more effective treatment are needed

Flunarizine is a drug developed to treat migraine [38]. It is used in AHC patients in China, Europe, Canada, and Japan, but not commonly used in the U.S. Flunarizine reduced the severity, duration, or frequency of the hemiplegic attacks in 68.3% of the patients, but could not lead to complete cure. Flunarizine is a non-selective calcium entry blocker targeting the CACNA family [39], not *ATP1A3*. There is an urgent need for a new drug to specifically target *ATP1A3*.

In summary, this study was the first genetic analysis performed in a Chinese AHC cohort. The *ATP1A3* mutation rate was 95.7% in our typical patients. We identified 13 novel missense mutations of *ATP1A3*. The majority of mutations were *de novo*. Genotype-phenotype correlation analysis showed that patients with epilepsy were more likely to carry E815K mutation. Logistic regression classifier exhibited accurate prediction on missense variant of whether it was neutral or predisposing for disease. Genetic testing of *ATP1A3* mutations is helpful for early diagnosis and confirming atypical cases of AHC.

## Supporting Information

Figure S1 **Protein sequence alignment of** ***ATP1A3*** **in the regions containing the novel mutations identified in our AHC patients.** Red shading highlights the 13 mutated residues. The arrow indicates the position of the mutations.
(TIF)

Figure S2 **Pedigree of one familial AHC and** ***ATP1A3*** **mutation identified in the family.** (A) Filled-in symbols indicate individuals with alternating hemiplegia of childhood, empty symbols indicate unaffected individuals, and symbols with a slash indicate deceased individuals. Arrow indicates the proband of the family. Individuals with *ATP1A3* mutation are indicated by m/+, and individuals of mutation-negative are indicated by +/+. (B) Chromatograms of *ATP1A3* mutation detected in the family. Arrows showed the position of the mutation. III-1 and II-1 were detected with the mutation c.2839G>C (p.G947R). II-2 was found to be negative with a wild type.
(TIF)

Table S1 **Collection of all variants on** ***ATP1A3*** **.** The ‘Source’ pointed out where the variant was gathered from. The ‘Position’ of insertion and deletion was the gap opening position at cDNA level. ‘1’ in ‘Transmembrane’ column meant the variant located at transmembrane domain which were defined according to Uniprot database. The ‘Frequency’ meant how many individuals carrying the variant.
(XLSX)

Table S2 **Molecular features investigated for** ***ATP1A3*** **missense mutations.** The value matrix of molecular features for each mutation. Full descriptions of each molecular feature was listed under the matrix.
(XLSX)

Table S3 **Correlation and discrimination effect between each molecular effect with mutation categories.** (A) Discriminative features for neutral variants *versus* disease mutations. (B) Discriminative features for AHC missense *versus* RDP missense. The ‘Spearman's rho’ was the spearman correlation between features with category, ‘Pvalue of term in logistic regression’ was the *P*-value of term in logistic regression between features with category, ‘Selected Frequency’ was the selected frequency at robust feature selection procedure, and ‘Feature class’ meant the class each feature belonging to.
(XLSX)
