# Supplementary figures and images for: ATP1A3 Mutations and Genotype-Phenotype Correlation of Alternating Hemiplegia of Childhood in Chinese Patients
Source: PLoS One. 2014 May 19;9(5):e97274. doi: 10.1371/journal.pone.0097274 (PMC4026576; doi:10.1371/journal.pone.0097274)

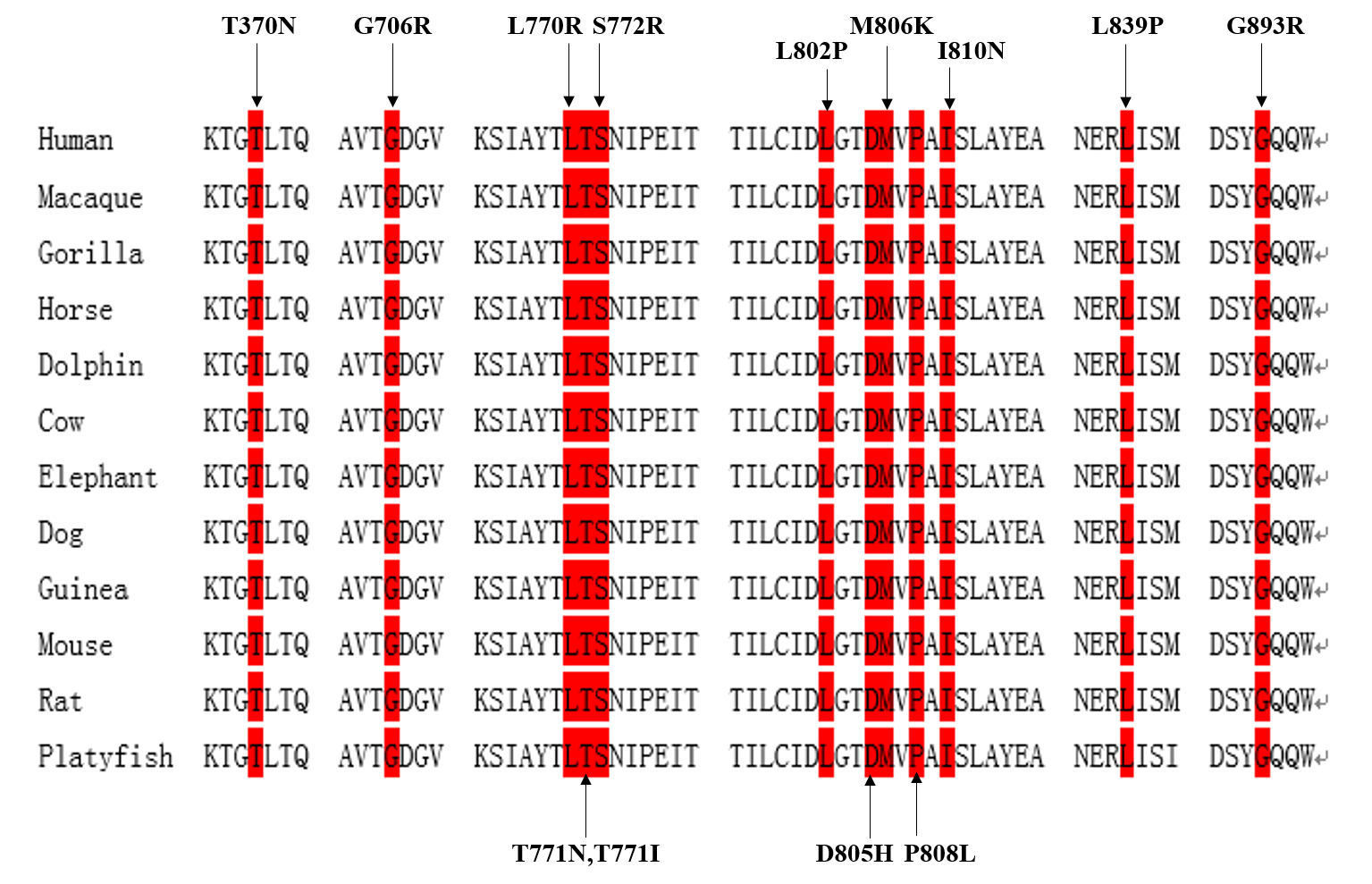

Supplement: Figure S1 — Protein sequence alignment of ATP1A3 in the regions containing the novel mutations identified in our AHC patients. Red shading highlights the 13 mutated residues. The arrow indicates the position of the mutations. (TIF) [file pone.0097274.s001.tif]

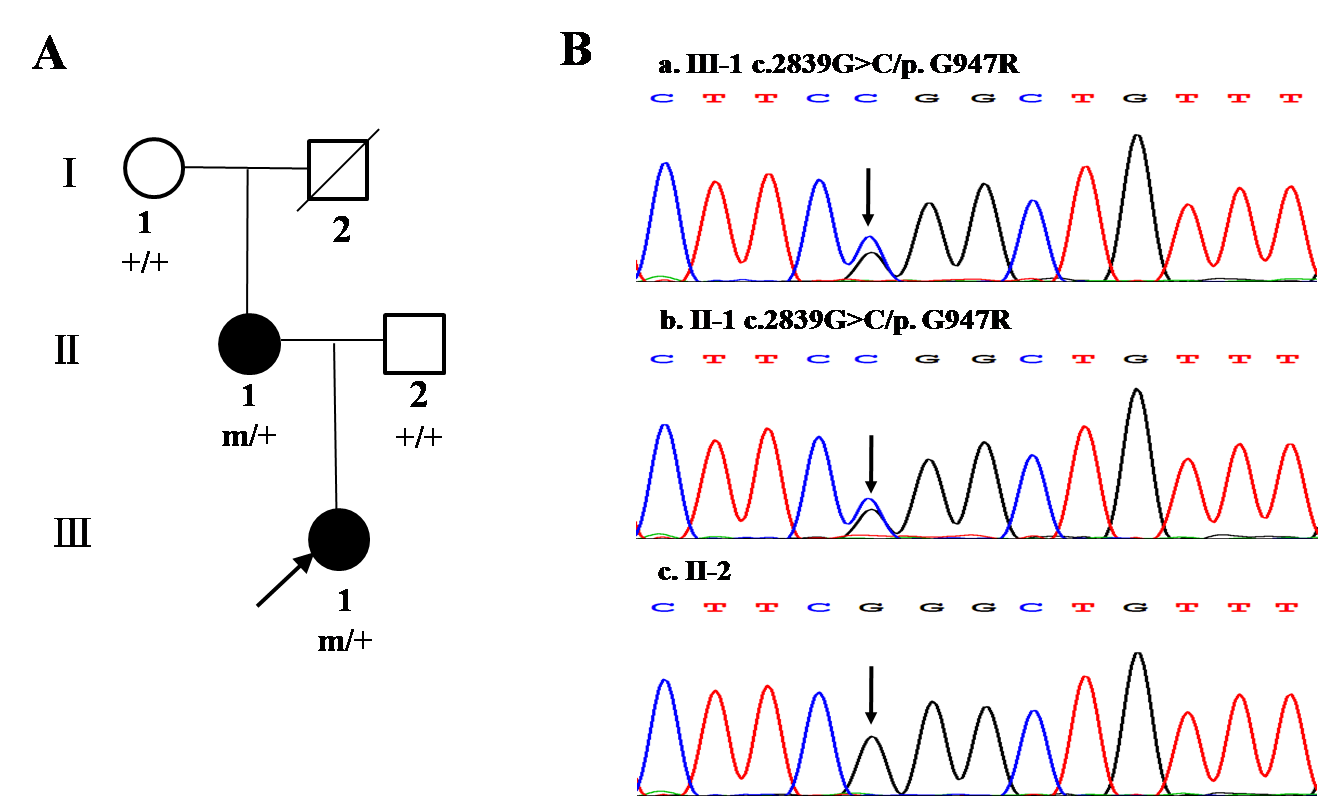

Supplement: Figure S2 — Pedigree of one familial AHC and ATP1A3 mutation identified in the family. (A) Filled-in symbols indicate individuals with alternating hemiplegia of childhood, empty symbols indicate unaffected individuals, and symbols with a slash indicate deceased individuals. Arrow indicates the proband of the family. Individuals with ATP1A3 mutation are indicated by m/+, and individuals of mutation-negative are indicated by +/+. (B) Chromatograms of ATP1A3 mutation detected in the family. Arrows showed the position of the mutation. III-1 and II-1 were detected with the mutation c.2839G>C (p.G947R). II-2 was found to be negative with a wild type. (TIF) [file pone.0097274.s002.tif]
